# The Primary Cilium: A New Player in Muscle Stem Cell Biology

**DOI:** 10.1007/s12015-026-11233-2

**Published:** 2026-09-12

**Authors:** Anna Rausch de Traubenberg, Ludivine Rotard, Caroline E. Brun

**Affiliations:** Institut NeuroMyoGène – Pathophysiology and Genetics of Neuron and Muscle (INMG-PGNM), CNRS UMR5261, Inserm U1315, Université Claude Bernard Lyon 1, Lyon, France

**Keywords:** Skeletal Muscle Regeneration, Muscle Stem Cell, Primary Cilium, Cilia-mediated Signaling, Quiescence, Fate

## Abstract

Adult muscle stem cells are responsible for skeletal muscle regeneration, a process where quiescent muscle stem cells activate, enter the cell cycle, and proliferate to give rise to either lineage-committed myoblasts for new muscle fiber formation, or self-renewing cells to replenish the stem cell reservoir. The regenerative capacity of muscle stem cells relies on their precise reception of environmental cues and appropriate intracellular responses. Quiescent muscle stem cells display a primary cilium, a microtubule-based organelle that protrudes from the cell surface and acts as a hub for chemical and mechanical signal transduction. Recent work has significantly increased our understanding of this organelle, demonstrating that the primary cilium actively regulates signaling pathways that are essential for maintenance of muscle stem cell quiescence, proliferation, and fate decision. In this review, we synthesize the current knowledge and recent advances on how this sensory organelle controls muscle stem cell quiescence, enables mechano-sensation, and regulates activation following tissue injury and during asymmetric self-renewal.

## Introduction

Developmental myogenesis is a stepwise process in which mesoderm-derived muscle progenitors proliferate and fuse into multinucleated, cylindrical and elongated muscle fibers (myofibers) to form skeletal muscle tissue [1]. Right before birth, muscle progenitors progressively adhere to myofibers and position themselves underneath the nascent basal lamina that surrounds each myofiber [2]. Due to this specific location, progenitors are referred to as satellite cells that all express the canonical Paired-box transcription factor PAX7 [3, 4]. After birth, satellite cells first proliferate to deliver new nuclei for postnatally growing myofibers and then, enter quiescence to constitute the adult muscle stem cell pool, around 8 weeks of age in mice [5]. Adult satellite cells or muscle stem cells (MuSCs) confer a robust regenerative capacity to the skeletal muscle and its postmitotic myofibers upon tissue damage [6].

**Fig. 1:**
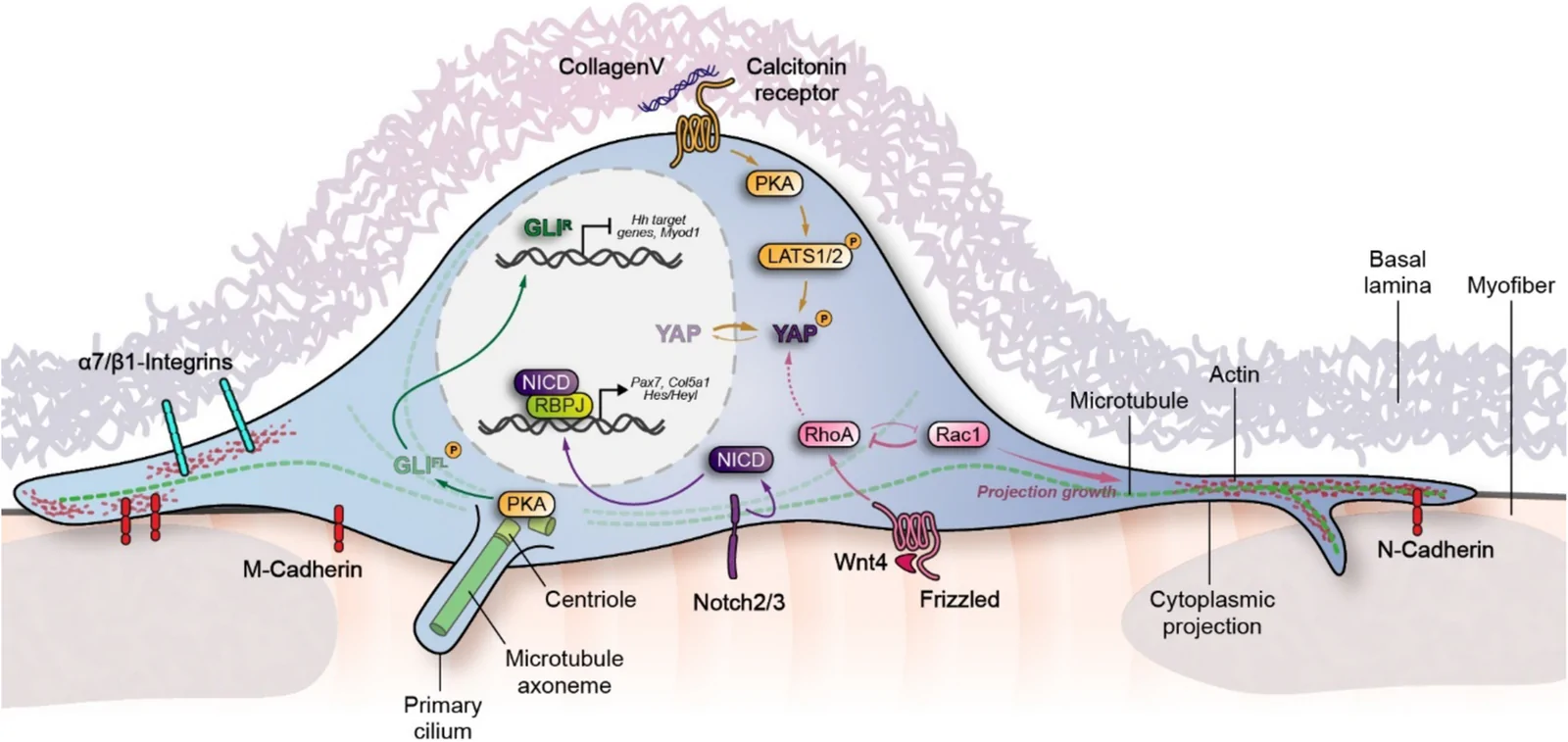
**Quiescence signaling of the muscle stem cell.** At steady state, Muscle Stem Cells (MuSCs) reside quiescent in their niche between the myofiber and the basal lamina. The quiescent MuSC is physically anchored to its niche by M- and N-Cadherins on the apical side, and α7/β1-Integrins on the basal side. Promoting adhesion to the niche, MuSC displays cytoplasmic projections that are constituted by a central core of microtubules surrounded by cortical actin and that are maintained by Rac1 activity [15]. Quiescent MuSC also has a primary cilium at the base of which ciliary protein kinase A (PKA) initiates the processing of GLI transcription factors into a repressive form that, in turn, inactivates Hedgehog pathway [11]. In addition, MuSC membrane is enriched for many signaling receptors that allow active sensing of the niche to promote quiescence [16]. For instance, upon binding to myofiber ligands Delta like (DLL) or Jagged (JAG), intracellular domains of Notch2/3 (NCID) are cleaved and translocate to the nucleus where they bind the transcription factor RBPJ and allow Notch target gene expression [17]. Among them is the *Col5a1* gene encoding CollagenV that signals in a cell-autonomous manner through the calcitonin receptor (CALCR) to maintain MuSC quiescence. CALCR detects CollagenV from the surrounding environment, leading to PKA activation and subsequent phosphorylation of LATS1/2 and YAP [18, 19]. Myofiber-secreted WNT4 binding to its receptor Frizzled activates RhoA signaling that decreases *Yap* mRNA expression, overall preserving quiescent MuSC tension and shape in the niche [20]. Therefore, balancing active and inactive RhoA is critical to maintain YAP inactive while allowing Rac1-dependent projection maintenance

**Fig. 2:**
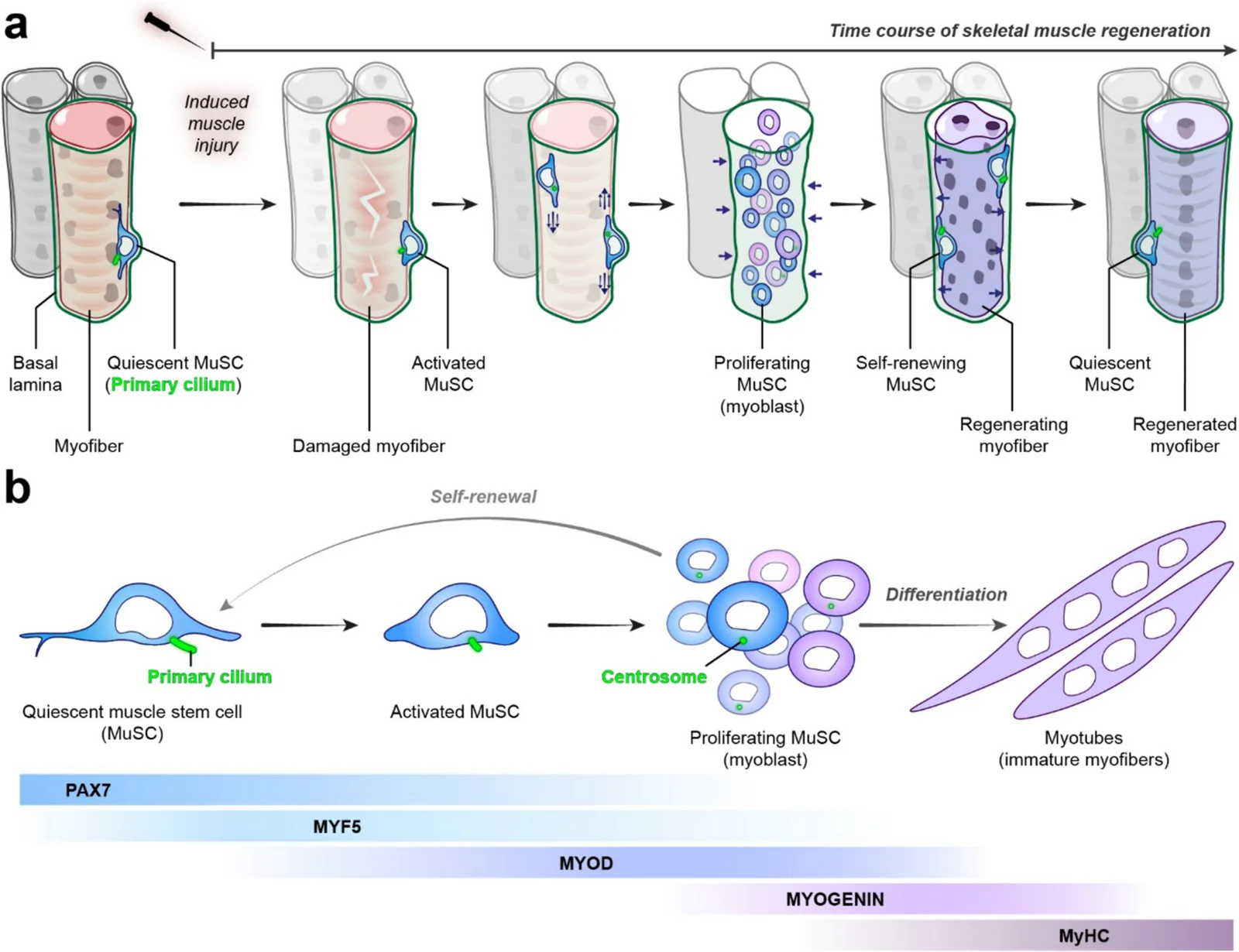
**Adult skeletal muscle regeneration.** **a)** Albeit quiescent in homeostasis, muscle stem cells (MuSCs) become activated (activated MuSCs) and enter the cell cycle upon muscle injury, giving rise to proliferating myogenic progenitors (or myoblasts) that will repair the tissue while maintaining the stem cell pool. MuSC behavior was exquisitely captured in vivo in mice by the laboratory of Gabrielle Kardon [21]. Upon activation, MuSCs migrate and proliferate extensively within the space vacated by the dying/dead fiber, also known as ghost fiber. Upon debris clearing by immune cells, ghost fibers progressively shrink triggering differentiation and fusion of most myoblasts into regenerated myofibers that display centrally located nuclei. In parallel, a pool of myoblasts exits the cell cycle and return to quiescence, in between the fiber and basal lamina, to replenish the stem cell pool **b)** Quiescent MuSCs possess a primary cilium that is disassembled as they activate, undergoes dynamic assembly/disassembly during proliferation, and is preferentially re-assembled on self-renewing MuSCs. Differentiated, multinucleated muscle cells, referred to as myotubes before maturing into myofibers, do not harbor a primary cilium. All MuSCs express the transcription factor PAX7. The myogenic regulatory factors MYF5 and MYOD commit MuSCs to the myogenic lineage, while MYOGENIN drives their differentiation and fusion, concomitantly with the expression of the Myosin Heavy Chains (MyHC) that will form the contractile units of the future myotubes

During homeostasis, adult MuSCs are quiescent and maintain a non-membrane-enclosed organelle called the primary cilium [7–13] (Fig. 1). In response to an injury, MuSCs activate, i.e., they exit quiescence and enter the cell cycle (Fig. 2a). This transition from quiescence to activation is accompanied by primary cilia disassembly [10–13]. Activated MuSCs proliferate extensively to generate lineage-committed progenitors, also called myoblasts, whose primary cilia are dynamically regulated, resorbing before mitosis and reassembling post-cytokinesis [10, 11]. Upon cell cycle exit, the majority of myogenic progenitors will differentiate and fuse to restore or form new myofibers that will ultimately regenerate the muscle tissue, whereas a subset will self-renew to replenish the stem cell pool (Fig. 2). Importantly, primary cilia specifically identify the self-renewing stem cell population, the terminally differentiated and fused progenitors do not display a primary cilium [10–12, 14].

**Fig. 3:**
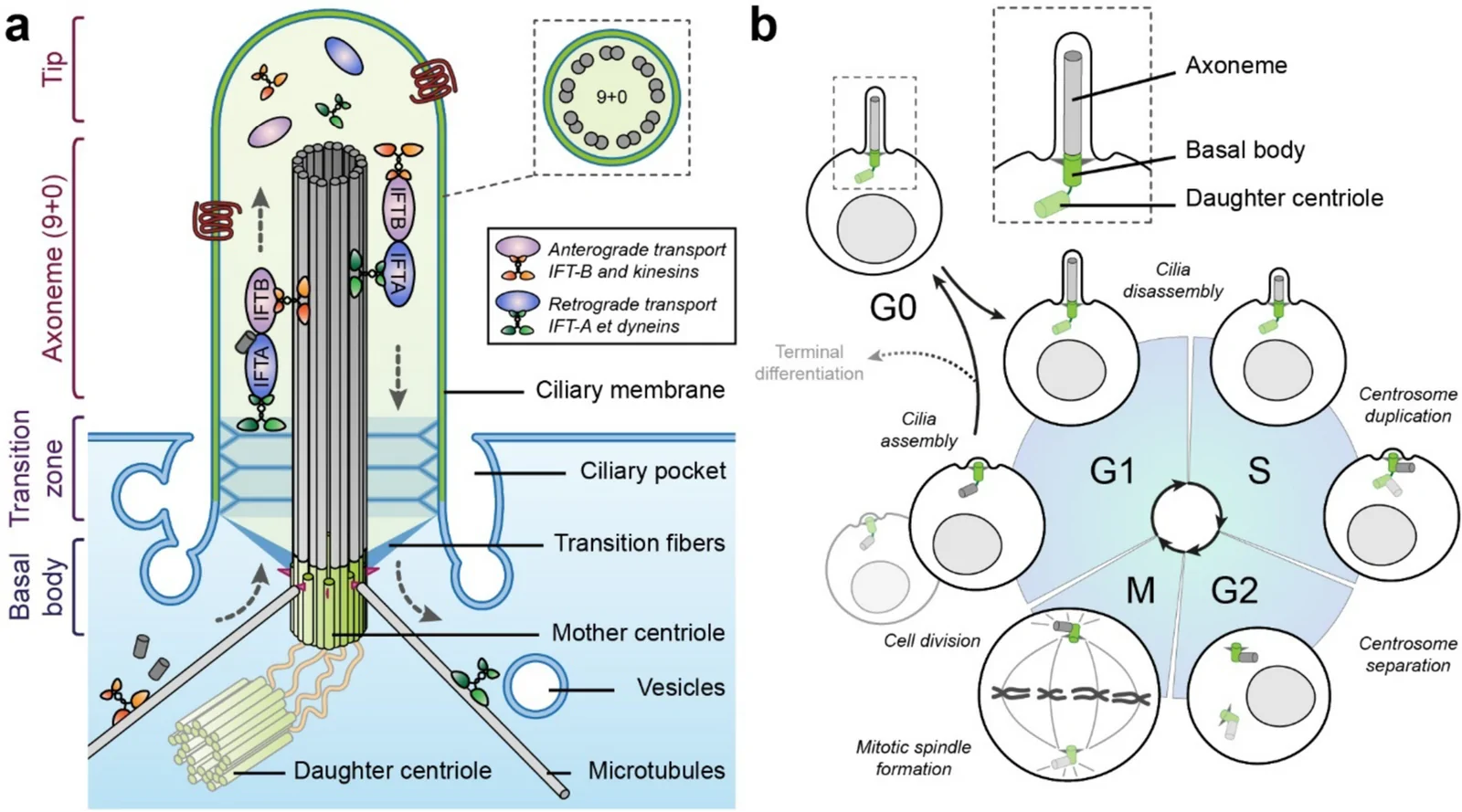
**Structure and assembly/disassembly dynamics of the primary cilium.** **a)** The primary cilium consists of three parts: the basal body, the transition zone, and the axoneme. The basal body is a modified centriole composed of nine triplets of microtubules (A, B, C). Only microtubules A and B extend to form a cylinder of nine peripheral microtubule doublets that constitute the transition zone and the axoneme with the specific (9 + 0) structure (*see* dotted box). Transition fibers connect the basal body to the plasma membrane. The ciliary membrane begins at the transition zone, where electron-dense projections (Y-shaped) connect the microtubules to the membrane. Together, they create a physical barrier – a molecular filter – separating the ciliary compartment from the rest of the cell. The intraflagellar transport (IFT) system maintains axonemal microtubules and facilitates the entry and exit of cilia proteins, receptors, etc. This anterograde transport is carried out by IFT-B proteins and kinesin molecular motors, while IFT-A and dyneins enable retrograde transport. **b)** The primary cilium is present in quiescent (G_0_) or differentiated cells and dynamically assembled and disassembled during the cell cycle. Cells in G_1_ possess a primary cilium that disassembles as they progress toward S phase, concomitantly with remodeling of the basal body. During S phase, centrosome duplicates in parallel of DNA replication, and the primary cilium is fully disassembled. In G_2_/M phase, centrosomes separate and form the mitotic spindles to ensure proper chromosome segregation into the two daughter cells during division. After mitosis, centrosomes can reassemble primary cilia, either in G_0_ or in G_1_ phase

While the first observation of a primary cilium-like structure on a muscle stem cell dates back to 1965 [22], its function has only recently begun to attract interest. The primary cilium is a microtubule-based structure that protrudes from the surface of almost all cells in vertebrates. It is anchored to a modified centriole, called the basal body, from which nucleates the axoneme, ensheathed by a ciliary membrane that is continuous with the plasma membrane [23] (Fig. 3a). The axoneme of the primary cilium is composed of nine doublets of microtubules, without central pair (“9 + 0” arrangement), whose posttranslational modifications, including acetylation and detyrosination, together with microtubule-associated proteins, contribute to regulating axoneme length and stability [24, 25]. Axonemal microtubules are also roamed by the intraflagellar transport (IFT) complexes carried by molecular motors, which form a dedicated transport machinery for molecules indispensable for cilia assembly and maintenance [26]. Ciliary protein import and export require cilia-targeting domains. Ciliary protein transport is controlled by the transition zone that forms above the basal body and acts as a physical barrier to prevent passive diffusion of cytoplasmic factors [27] (Fig. 3a). The transition zone thus separates the primary cilium from the rest of the cell, making it a unique organelle in terms of membrane and ‘cilioplasm’ composition.

The ciliary membrane constitutes a specialized surface of exchange with the environment and the cilioplasm concentrates effectors and molecules of signaling pathways [27, 28]. The primary cilium is therefore often compared to a cellular antenna acting as signaling hub. The composition, length, spatial positioning and assembly/disassembly of primary cilia are dynamically and temporally regulated, enabling fine-tuned control of cilia-mediated signaling [29]. In fact, emerging data, including cilia-targeted proteomics, revealed cell-type specificity of ciliary composition, suggesting tissue-specific signaling functions [30, 31]. Even within a given cell type, primary cilia can redistribute their molecular content within seconds after mechanical or chemical stimulation, allowing a swift modulation of signaling pathways [32].

It is also assumed that variation of cilia length acts either by concentrating factors in a smaller volume or by increasing the exchange surface with the surrounding environment [33], while the cellular positioning of the primary cilium promotes ligand binding or mechanical sensing in specific tissue compartments [34]. For instance, cilia length and orientation in the brain follow specific rhythmic fluctuations during the dark/light phases of the day, suggesting a structured spatiotemporal organization that could contribute to circadian-driven processes [35]. In neurons, primary cilia are assembled in the leading process to regulate the polarity and directionality of the migrating cells [36]. In renal epithelial cells, primary cilia project from their apical side into the tubular lumen, thereby sensing fluid flow [37].

Finally, the dynamics of cilia assembly and disassembly contribute to regulating cilia-driven signal transduction [23]. Cilia remodeling is controlled by a wide range of enzymes, many of which are also associated with mitosis [38]. These include microtubule-depolymerizing kinesins KIF2A, KIF19A, KIF24; the mitotic Aurora kinase A (AURKA) and Polo-like kinase 1 (PLK1); associated proteins such as HEF1; and the Histone Deacetylase 6 (HDAC6) that drives destabilization of axonemal microtubule doublets [39]. Indeed, ciliogenesis and cilia resorption are intimately linked to cell cycle progression [38]. Because of high cytoskeleton remodeling and centrosome mobilization to organize the mitotic spindles, cilia and mitosis are mutually exclusive. Primary cilia are disassembled in two steps starting at the G_1_/S transition and right before the mitosis onset, and reassemble when cells exit mitosis either to enter G_1_ phase, G_0_, or to terminally differentiate [27] (Fig. 3b). In vitro, primary cilium assembly is promoted by serum starvation, which triggers cell cycle arrest in a variety of cell lines (3T3, RPE1, C2C12), leading to the axiom that primary cilium is a hallmark of quiescence [8, 38–40]. As quiescent stem cells are often physically constrained in their niche, validating their ciliation status in situ is more challenging but has been demonstrated in several cell types, such as neural stem cells, fibro-adipogenic progenitors (FAPs) and muscle stem cells.

In the following sections, we discuss the uncovered functions of primary cilia for the homeostatic maintenance of muscle stem cell quiescence in adult skeletal muscle and for the regulation of their activation, proliferation and fate during regenerative myogenesis.

## Primary Cilia Retain Muscle Stem Cell Quiescence

In homeostasis, the muscle stem cell (MuSC) resides quiescent in its niche, in between the myofiber and the basal lamina [6, 41, 42] (Fig. 1). Quiescence is crucial for the long-term maintenance of the MuSC reservoir as it protects cells from replicative and metabolic stresses, thus preventing stem cell exhaustion [43]. It is a reversible state of cell cycle arrest, in which cells show low RNA and protein contents, as well as reduced metabolic activity [44, 45]. Yet, quiescent MuSCs exhibit dynamic transcriptional activity and effective translational control, priming them for a rapid response to extrinsic stimuli [44–49]. Maintaining quiescence is thus a highly active process involving several signaling pathways that primarily repress the myogenic program and cell cycle entry while preserving cellular interactions with the niche.

Quiescent MuSCs develop long and elaborated, microtubule-based cytoplasmic projections that run on the myofiber, thus expanding their surface area for interaction with the niche [15, 50]. Additionally, they often display a primary cilium on the apical side of the cell, facing the myofiber [7, 10, 11, 51]. These two microtubule-based structures, namely the quiescent projections and the primary cilium, are features of quiescent MuSCs (Fig. 1), as both disassemble upon activation. They are likely acting as sensors to survey any change within their micro-environment and to control the quiescence-to-activation MuSC transition [11, 52].

Anne-Gaelle Borycki’s laboratory pioneered the quantitative analysis of quiescent MuSCs harboring a primary cilium [10]. First analyses on histological cross-sections of muscle revealed that fewer than half of quiescent MuSCs were observed with a primary cilium [9, 10]. This low proportion may stem from technical limitations for morphometric analysis of MuSCs on sections, owing to the constrictive niche environment (compressed by the basal lamina against the myofiber), and the section thickness that does not allow imaging of the entire cell (MuSC size including the projections can reach up to 80 μm [15, 50]). Ex vivo preparations, such as single myofiber isolation, greatly improved MuSC visualization from their gross morphology to their intracellular composition. On freshly isolated myofibers from the *extensor digitorum longus* muscle, three quarters of quiescent MuSCs display a primary cilium [7, 10, 11]. Nonetheless, muscle dissociation into single myofibers requires partial digestion of the basal lamina and the MuSC niche, inevitably leading to stem cell activation [44, 49]. It may thus be reasonable to think that the proportion of ciliated MuSCs is higher in situ.

The primary cilium is quickly disassembled after activation [10, 11, 13]. Accordingly, blocking cilium disassembly was shown to prevent MuSC activation [13]. Treatment with Tubastatin A (TubA), a specific HDAC6 inhibitor, impedes cilia disassembly and allows maintaining a higher proportion of ciliated MuSCs post-isolation [13, 39, 53]. Ex vivo TubA-treated MuSCs do not enter the cell cycle and retain quiescent features, such as low RNA content and low expression of MYOD that is among the first myogenic regulatory factors translated upon MuSC activation [13, 45] (Fig. 2b). Accordingly, they display decreased expression of cell cycle-promoting genes. However, the transcriptomic signatures of ex vivo cultured MuSCs treated by TubA and freshly isolated MuSCs do not overlap [13]. This indicates that HDAC6 inhibition blocks cilia disassembly but fails to fully maintain quiescence. It also suggests that the primary cilium alone is not sufficient to define MuSC quiescence.

## Cilia-mediated Signaling Contributes to Keep Muscle Stem Cell Quiescent

### Ciliary Hedgehog Signaling in Quiescent Muscle Stem Cells

The primary cilium participates in MuSC quiescence maintenance by controlling at least the Hedgehog pathway. Hedgehog signaling remains the only canonical pathway absolutely requiring primary cilia for its transduction that relies on the GLI family of three transcription factors [54–56]. The GLI proteins follow a specific code to cooperate in regulating cell fate and stemness [57, 58].

**Fig. 4:**
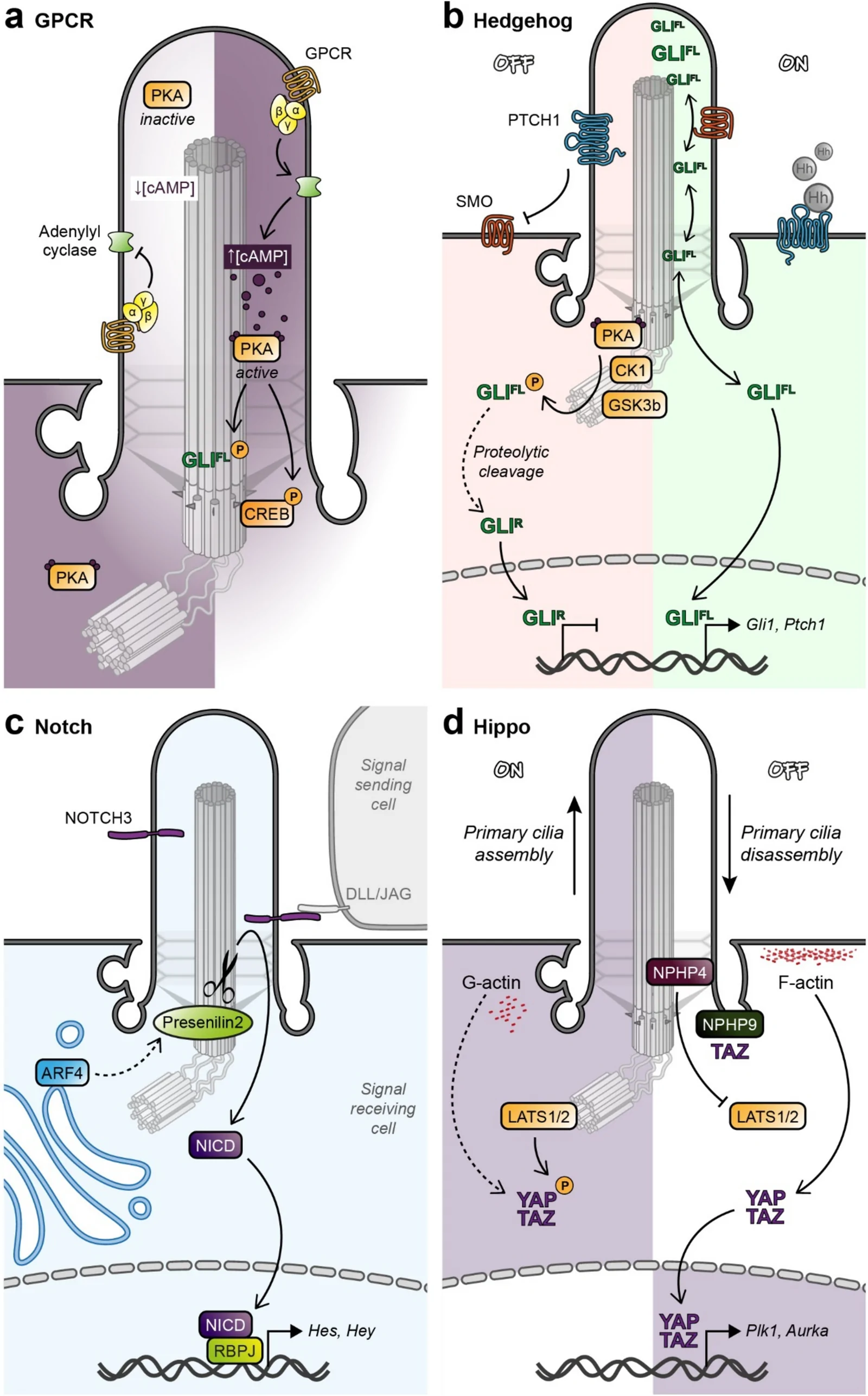
**Cilia-mediated signaling pathways. a)** Compared to the cytoplasm, the primary cilium exhibits a vast surface of exchange combined to a small internal volume. This geometry results in an increased surface-to-volume ratio that can improve the efficiency of ciliary effectors, as it has been modeled for cyclic adenosine monophosphate (cAMP)-dependent pathway [60]. Ciliary G-protein-coupled receptors (GPCRs) interact with G proteins at the membrane. Upon ligand binding, GPCRs undergo a conformational change, freeing the G proteins that subsequently activate the adenylyl cyclases. The adenylyl cyclases then catalyze the conversion of ATP into cAMP, which increases ciliary cAMP concentration, local activation of the protein kinase A (PKA) and cAMP response element-binding protein (CREB) phosphorylation [61]. Compartmentalization of PKA activity also permits GLI processing, thus controlling Hedgehog pathway [60]. **b)** The Hedgehog (Hh) pathway is a cilia-driven signaling. In absence of Hh ligand, full-length GLI (GLI-FL) transcription factor is phosphorylated at the base of the cilium by the PKA, the glycogen synthase kinase 3β (GSK3β), and the casein kinase 1. This induces GLI proteolytic cleavage and release into a truncated and repressive form (GLI-R) that inhibits Hh target gene expression. In presence of Hh ligands, the receptor Patched1 (PTCH1) exits the primary cilium while the orphan GPCR Smoothened (SMO) enters it. SMO ciliary enrichment prevents GLI-FL proteolytic cleavage, allowing GLI-FL to accumulate into the primary cilium, and subsequently to translocate in the nucleus to promote Hh target gene expression. **c)** The HIPPO signaling relies on the two central effectors YAP/TAZ. **(ON)** When the HIPPO pathway is activated, YAP/TAZ are phosphorylated by kinases, such as LATS1/2, which prevents YAP/TAZ nuclear translocation. **(OFF)** Non-phosphorylated YAP/TAZ can then translocate to the nucleus and bind TEAD transcriptional factor to activate target gene expression. Among the target genes are *Polo-like kinase 1* (*Plk1*) and *Aurora kinase A* (*Aurka*) that trigger primary cilia disassembly. Additionally, the ciliary protein Nephrocystin 4 (NPHP4) can inhibit LATS1/2-mediated phosphorylation of YAP/TAZ, while Nephrocystin 9 (NPHP9) hinders TAZ cytoplasmic retention, both contributing to inactivating HIPPO signaling. The ratio of F to G actin also regulates YAP/TAZ activity via kinases such as LIMK or by inducing cell rounding [62, 63]. **d)** In the developing epidermis, ciliary NOTCH3 drives cell differentiation. In differentiating epidermal cells, the small GTPase ARF4 controls Presenilin2 targeting to the basal body. Therefore, it allows spatial regulation of Notch signaling at the primary cilium, which is required for proper cell differentiation

In the absence of ligand, GLI2 and GLI3 transcription factors are sequentially phosphorylated at the base of the primary cilium by the cAMP-dependent protein kinase A (PKA), the glycogen synthase kinase 3β (GSK3β), and the casein kinase 1 (CK1). This triggers GLI ubiquitination and proteolytic cleavage by the proteasome (Fig. 4a, b). The proteolytically processed forms of GLI proteins, mostly GLI3, repress the expression of Hedgehog target genes [56, 57]. However, upon ligand-binding to the Hedgehog receptor Patched1 (PTCH1), the G protein-coupled receptor Smoothened (SMO) accumulates in the primary cilium, which blocks GLI proteolytic processing and allows GLI conversion into full-length transcriptional activators. These activators accumulate in the cilioplasm and at the tip of the primary cilium before translocating to the nucleus, where GLI2 primarily promotes the expression of *Gli1*, *Ptch1*, and other Hedgehog target genes [55, 57]. The classical GLI code thus assumes that GLI factors act as transcriptional repressors by default in the absence of Hedgehog ligands, preventing the expression of downstream target genes. However, recent work revises this model and proposes that GLI-mediated repression is a timely regulated process that is only switched on in a context-dependent manner [58, 59]. As such, loss of GLI factors does not automatically lead to de-repression of Hedgehog target genes in the absence of signals.

As observed in hematopoietic or hair follicle stem cells, cilia-mediated repression of Hedgehog activity in MuSCs associates with quiescence [64–66]. Indeed, in quiescent MuSCs, PTCH1 is not expressed and GLI3 is expressed at the base of the primary cilium, where it colocalizes with PKA [11, 66]. PKA-mediated GLI3 phosphorylation precedes its processing into the repressive GLI3R form [11, 66]. Remarkably, specific deletion of the PKA ciliary target *Gli3* in MuSCs does not trigger Hedgehog target gene upregulation and MuSC activation [11] (Table 1). Instead, it induces a transition from a deep to a mTOR-dependent ‘alert’ state of quiescence. Although quiescent MuSCs were long considered fixed in a unique, dormant G_0_ phase of minimal activity, a growing body of evidence converges on the theory that quiescence comprises multiple functional states [52, 68]. This concept emerged with the description of a G_Alert_, a reversible ‘alert’ phase in which G_0_ stem cells respond to systemic signals released from a distant injury, thereby priming quiescent MuSCs for rapid activation without disruption of the niche or immediate cell cycle entry [11, 47, 69, 70]. Consistent with this model, *Gli3*-deficient quiescent MuSCs do not enter the cell cycle, maintain a primary cilium, and transition to G_Alert_, independently of tissue damage-associated signals [11]. More recently, it was reported that, in homeostatic muscle, *Gli1* expression identifies a small subset of quiescent MuSCs that do not express *Gli3* and exhibit features of G_Alert_ [71]. Although *Gli1* itself is dispensable in MuSCs and solely functions as a marker [72], *Gli1* + cells were proposed to act as a sentinel subpopulation of quiescent MuSCs, poised for rapid activation and differentiation in response to muscle injury [71].

**Table 1:** Lessons from genetic mouse models

Floxed gene	Function	Stage- and lineage-specific deletion	Muscle phenotype
*Talpid3*	Basal body protein required for primary cilia assembly	Adult *Pax7*-expressing muscle stem cells	Decreased MuSC proliferation Decreased MuSC self-renewal Impaired muscle regeneration [12]
*Ift88*	IFT protein required for primary cilia assembly and maintenance	Adult *Pax7*-expressing muscle stem cells	Decreased MuSC proliferation Decreased MuSC self-renewal Impaired muscle regeneration [7]
		Embryonic *Pax7*-expressing muscle stem cells	Decreased body weight, hydrocephalus, inability to stand [67]
*Arl3*	Ciliary membrane protein regulating protein trafficking	Embryonic *Myf5*-expressing muscle progenitors	Decreased exercise-induced muscle hypertrophy [67]
		Embryonic *Pax7*-expressing muscle stem cells	Decreased exercise-induced muscle hypertrophy [67]
*Gli3*	Transcription factor of Hedgehog signaling	Adult *Pax7*-expressing muscle stem cells	Faster MuSC activation Increased MuSC proliferation Increased MuSC self-renewal Enhanced muscle regeneration [11]
*Smo*	Co-receptor of Hedgehog signaling	Adult *Pax7*-expressing muscle stem cells	Delayed MuSC activation Longer MuSC cell cycle Decreased MuSC self-renewal Impaired muscle regeneration [66]
*Ptch1*	Receptor of Hedgehog signaling	Adult *Pax7*-expressing muscle stem cells	Faster MuSC activation Shorter MuSC cell cycle Increased MuSC self-renewal Impaired muscle regeneration [66]

### Putative Cilia-mediated Signaling Pathways in Quiescent Muscle Stem Cells

Altogether, these results reinforce the idea that quiescence encompasses several states with heterogeneous functional properties, which are in part governed by ciliary Hedgehog signaling in MuSCs. It is therefore conceivable that additional cilia-mediated mechanisms contribute to the diversity of MuSC quiescence states beyond the Hedgehog signaling. For instance, Notch pathway actively sustains MuSC quiescence through a Collagen V (COLV)-induced activation of the Calcitonin receptor (CALCR) signaling cascade [18, 73] (Fig. 1). Whether Notch activity involves MuSC primary cilium is unknown. However, it is noteworthy that several Notch signaling components localize in the primary cilia of suprabasal epidermal cells, where they fine-tune the balance between proliferation and differentiation [74] (Fig. 4c).

In quiescent MuSCs, the Notch/COLV/CALCR cascade activates PKA that phosphorylates the Hippo kinases LATS1/2 and eventually, represses YAP activity [18, 19, 75]. Conversely, YAP/TAZ phosphorylation is greatly reduced in cycling MuSCs, allowing YAP/TAZ nuclear translocation and the expression of PAX7 and MYOD, which promote cell proliferation [76, 77]. Regulating a plethora of cellular processes during development, homeostasis and regeneration [78], the Hippo pathway also modulates MuSC quiescence by maintaining their tension, shape and retention within the niche [19, 20, 79]. Hippo induction by molecular/mechanical stimuli recruits kinases that, ultimately, phosphorylate the two transcription factors YAP and TAZ, preventing their nuclear translocation and the expression of downstream target genes (Fig. 4d). In addition to COLV-mediated inhibition of YAP activity, other signals from the niche contribute to the control of MuSC stiffness and quiescence. For instance, the myofiber-secreted WNT4 enhances RhoA-dependent suppression of *Yap* mRNA, thereby blocking cell cycle entry [20] (Fig. 1).

Given that primary ciliogenesis and Hippo activation share similar extracellular stimuli and dynamics during muscle regeneration, one could speculate that their functions are interconnected in MuSCs, all the more so that reciprocal interactions between Hippo signaling and primary cilia assembly are well established [80]. Indeed, non-phosphorylated nuclear YAP/TAZ promotes the expression of the mitotic kinases *Aurka* and *Plk1* and, consequently, the disassembly of cilia, while YAP/TAZ phosphorylation and cytoplasmic retention favors ciliogenesis [62]. Conversely, the ciliary proteins Nephrocystin 4 and 9 (NPHP4, NPHP9) inhibit the Hippo pathway, NPHP4 preventing LATS1-mediated phosphorylation of YAP/TAZ, and NPHP9 stabilizing TAZ in the nucleus [81, 82] (Fig. 4d). Consistently, both ciliogenesis and Hippo signaling are associated with cell cycle-dependent cytoskeletal rearrangements, particularly actin stability and dynamics [83]. Upon activation, MuSCs undergo drastic cytoskeleton remodeling – including RhoA-mediated retraction of their cytoplasmic projections, primary cilium disassembly and morphological rounding – to facilitate cell cycle entry [10, 11, 15, 20]. Together, these cilia-associated signaling cascades – spanning Hedgehog repression, Hippo-mediated YAP/TAZ inhibition, and RhoA-regulated cytoskeletal dynamics – position the primary cilium as a putative mechanosensory hub for controlling MuSC quiescence heterogeneity and activation.

## Primary Cilia Enable Muscle Stem Cells to Sense the Myofiber

### Ciliary Mechanotransduction

Mechanical environment is a critical driver of stem cell behavior [84]. For instance, a compressive environment favors MuSC quiescence [85, 86], while an increase in substrate stiffness promotes proliferation and differentiation [87, 88]. Cells perceive mechanical cues from their surrounding microenvironment through membrane structures (e.g., the adherens junctions, focal adhesions, mechanoreceptive ion channels, or primary cilium), converting them into biochemical signals.

First evidenced in renal epithelial cells where the deflection of primary cilia by the luminal flow triggers a calcium influx [89], the mechanosensory role of the primary cilium is now established in a diversity of cell types, particularly in tissues that are often subjected to mechanical stimulation, such as the musculoskeletal system [90]. In bone, the osteoblasts, the osteocytes and the mesenchymal stromal cells are ciliated and respond to loading-induced fluid flow in a cilia-dependent manner [91]. For instance, fluid flow stimulation induces Adenylyl Cyclase 6 (ADCY6)-dependent expression of osteogenic genes in human bone marrow-derived mesenchymal stem cells in vitro [92, 93]. In osteocytes, siRNA-mediated knockdown of either *Ift88*, which abrogates primary cilia assembly, or the ciliary enzyme *Adcy6*, reduces loading-induced bone formation [90]. In cartilage, chondrocyte matrix deposition induced by loading also relies on primary cilia [94]. Interestingly, in chondrocytes, primary cilia are enriched for extracellular matrix-binding proteins, suggesting that they are sensitive, not only to fluid shear stress, but also to surrounding tissue deformation [94, 95]. In tendons, about two-thirds of tenocytes have primary cilia that are aligned with the collagen fibers, and cilia length is dynamically regulate during mechanical loading or stress removal [96–98].

### Primary Cilia-dependent Mechanosensation in Muscle Stem Cells

To date, the mechanosensory role of the primary cilium in muscle stem cells (MuSCs) remains poorly understood. In homeostatic muscle, primary cilia are commonly observed on the apical side of the MuSCs, positioning them ideally to sense myofiber-derived mechanical cues [7, 11, 12]. This orientation is reminiscent of what is observed in kidney epithelial cells and endothelial cells, where apical primary cilia project towards the fluid flow and function as mechanosensors [37, 99].

In 2022, Li et al. uncovered that MuSC primary cilium acts as a mechanosensor in the context of exercise-induced muscle atrophy in mice [67]. Following stretching-induced mechanical stimulation of mouse hindlimb muscles or in vitro primary myoblasts, they observed increased expression of the myogenic regulatory factors *MyoD* and *Myogenin*, as well as the differentiation marker *Myosin Heavy Chains* (MyHC). Mechanical stretching also upregulates the Hedgehog-related genes *Ptch1* and *Gli1*. Importantly, primary cilia of mechanically stressed myoblasts show an enrichment of GLI3 in the cilioplasm and at the cilia tip, suggesting that mechanical stimulation abolishes GLI3 proteolytic conversion into the transcriptional repressor form and promotes myogenesis through cilia-mediated Hedgehog activation [67]. Consistent with this hypothesis, this pro-myogenic effect is abrogated upon cilia disruption.

To disrupt primary cilia in vivo, the authors used different Cre/Lox mouse models in which *Arl3* deletion compromises ciliary function in either the *Pax7*-expressing MuSCs and their progeny, or in the *Myf5*-expressing cells, targeting the myogenic lineage [4, 100, 101] (Table 1). The mutant mice show little to no improvement after training and do not gain muscle mass, supporting a functional requirement for MuSC primary cilia in exercise-induced muscle hypertrophy. However, whether this phenotype reflects a specific defect in MuSC primary cilia mechanosensing or is secondary to alterations in the MuSC pool remains unclear, as PAX7+ MuSC numbers were not assessed in any of these models. Consequently, it is difficult to fully attribute the observed in vivo defects solely to impaired cilia signaling, irrespective of potential changes in the stem cell compartment having occurred during development.

Yet, this study provides the first evidence of a ciliary mechanosensation in myogenic progenitors in vitro and raises the exciting hypothesis that mechanical stimulation could act as a trigger of Hedgehog transduction. Although mechano-induced Hedgehog signaling remains broadly unexplored, it recently emerged alongside canonical mechanotransduction pathways in several cell types, in the context of fibrosis and ligamentum flavum hypertrophy for instance [102]. This work also opens new questions regarding primary cilia function during muscle development. Indeed, disrupting cilia function by knocking-out *Arl3* in MuSCs and myogenic progenitors has no major effect on muscle development and mice grow normal. However, knocking-out *Ift88* to prevent ciliogenesis in the *Pax7*-expressing cells results in a severe phenotype that includes lower body weight, hydrocephalus and inability to stand, partly phenocopying the *Pax7*^−/−^ mice [4, 67, 103, 104] (Table 1). This suggests that the presence of a primary cilium, even non-fully functional, is required for a proper muscle development and likewise contributes to the establishment of the adult muscle stem cell pool.

## Primary Cilia Control Muscle Stem Cell Proliferation and Safeguard Self-renewal

### Cell Cycle-dependent Assembly and Disassembly of the Primary Cilium

Upon appropriate stimuli, muscle stem cells (MuSCs) transition from quiescence to activation, progressively disassembling their primary cilium and undergoing robust proliferation (Fig. 2) [6]. Ciliary dynamics during proliferation have been analyzed in C2C12 myoblast cell line, primary murine MuSCs, and human myogenic progenitors [7, 10, 11, 14, 51]. Depending on the model, 5–25% of proliferating myoblasts transiently exhibit a primary cilium [8, 11]. This discrepancy partly reflects differences in cell type doubling times, as ciliogenesis follows the cell cycle dynamics. Indeed, mitosis triggers a dramatic remodeling of the microtubule cytoskeleton, duplication and repurposing of the centrosomes for the mitotic spindle assembly, which requires cilia disassembly and complete absence during the S and the G_2_-M phase of the cell cycle, respectively (Fig. 3b). Hence, cilia are only assembled and visible in G_1_ phase. As expected, the proportion of ciliated MuSCs increases when cells are blocked in G_1_ phase by a treatment with CDK4/6 inhibitor, but dramatically decreases upon withdrawal [10].

Despite their transience, primary cilia are essential for MuSC proliferation. Although forcing cilia assembly blocks the cell cycle progression, removing the primary cilium does not promote the cell cycle, but rather dampens it. Blunting primary cilia formation *via* MuSC-specific conditional mouse knockout of *Ift88* or *Talpid3* – a centrosomal protein required for the basal body docking at the apical side of the cell [105] – dramatically decreases the expression of cell cycle promoting genes and the overall proliferative capacity of MuSCs and their descendant myogenic progenitors [7, 12] (Table 1). As a consequence, acute injury-induced muscle regeneration in mice induces self-renewing MuSC depletion, smaller regenerated fibers, and diminished muscle force. This was further supported by in vitro studies where *Ift88* silencing impairs C2C12 myoblast proliferation, overall reducing their ability to progress through the cell cycle or to differentiate [8, 51]. Importantly, serial muscle injuries aggravate the phenotype in vivo [12], indicating that disrupting ciliogenesis does not only hinder proliferation but also biases MuSCs towards differentiation, overall reducing their reservoir and regenerative capacity. Cell cycle exit occurs either permanently in senescent or differentiating post-mitotic cells, or reversibly when cells return to quiescence. With a few exceptions [106], cells reassemble a primary cilium upon cell cycle exit. In mouse skeletal muscle, primary cilia preferentially reassemble on the self-renewing MuSCs [10–12, 14]. It is therefore not surprising that drug-mediated disruption of the primary cilium reassembly following cell division disrupts MuSC ability to self-renew at the expense of differentiation [10].

### Cell Cycle-dependent Dynamics of Ciliary Signaling

Mechanistically, ciliary Hedgehog signaling controls cell proliferation. Indeed, in proliferating myoblasts, GLI3 accumulates in the primary cilium and is mainly released as the full-length form (GLI3FL) rather than the GLI3R repressive form [11, 51, 67]. The increased fraction of GLI3FL over GLI3R diminishes its repressive activity, thus de-repressing Hedgehog target genes and promoting cell proliferation [7, 11]. Consistently, mice treated with the Hedgehog agonist SAG (Smoothened Agonist) that abrogates GLI3 proteolytic processing, or Hedgehog signaling gain-of-function mouse models display increased MuSC proliferation during induced-skeletal muscle regeneration [7, 11, 66]. Importantly, Hedgehog pro-proliferative signaling requires primary cilia for transduction, as *Ift88*^−/−^ MuSCs and myogenic progenitors fail to respond to Hedgehog stimulation [7, 11, 67]. Despite the transience of this signaling organelle, the activity of Hedgehog effectors outlasts the disassembly of the primary cilium in G_2_-M and continues towards the next G_1_ phase of the cell cycle [107]. Whether the association between primary cilia and cell cycle signaling goes beyond the Hedgehog pathway remains to be determined in MuSCs. Yet, negative regulation of the cell cycle by the primary cilium in particular, is quite a strategic topic of study insofar as it contributes to the regulation of cell cycle exit, thus driving cell fate.

Muscle injury triggers MuSC proliferation to produce thousands of lineage-committed progenitors that will primarily differentiate and fuse into syncytia, resulting in multinucleated and non-ciliated myofibers [1] (Fig. 2). In parallel, a portion of these progenitors will self-renew and reconstitute the stem cell reservoir. Interestingly, cilia-mediated Hedgehog signaling participates in MuSC fate decision [7, 11, 12, 66]. Upon cell cycle exit, GLI3 no longer accumulates in the primary cilium of cells primed to self-renew and undergoes proteolytic cleavage that progressively induces Hedgehog repression [11]. Hedgehog signaling shutdown will ultimately allow MuSCs to return to quiescence [66]. Thus, Hedgehog activation indirectly induced by either knocking-out *Ptch1* or *Gli3*, favors MuSC expansion and self-renewal [11, 66], whereas Hedgehog loss-of-function through MuSC-specific knock-out of *Smoothened* (*Smo*) results in a decreased number of self-renewing cells, detrimental for the muscle long-term regenerative capacity [66] (Table 1). These findings parallel observations in adult neural stem cells of the subventricular zone, where long-term Hedgehog activation induces an accumulation of neural stem cells concomitant with diminished neurogenesis [108]. Even though both *Ptch1*^−/−^ and *Gli3*^−/−^ MuSCs have increased activation of Hedgehog signaling, their regenerative capacities do not entirely overlap. While *Ptch1*^−/−^ MuSCs undergo a transient increase in progenitor cell proliferation during muscle regeneration [66], *Gli3*^−/−^ MuSCs sustain superior proliferation levels overtime, which impedes proper return to quiescence [11]. These results suggest that the receptor PTCH1 and the transcription factor GLI3 are implicated in roles other than the cilia-mediated Hedgehog transduction. Supporting this hypothesis, *Gli3*^−/−^ MuSCs exhibit increased activation of mTOR signaling [11]. Single cell transcriptomics also revealed that MuSC-specific *Talpid3*-knockout impairs Hedgehog and WNT signaling [12]. Interestingly, in *Talpid3*^−/−^ MuSCs, activation of WNT signaling by drug-mediated GSK3β inhibition restores muscle regeneration, whereas pharmacological stimulation of Hedgehog signaling has no effect. Thus, WNT activation bypasses the lack of functional primary cilium to restore MuSC function.

Together, these data indicate that cilia-driven MuSC fate decision includes pathways other than Hedgehog. As a matter of fact, the ability of the primary cilium to regulate cell fate by driving distinct signaling pathways, allowing for their spatiotemporal control, has already been demonstrated in the context of epidermal differentiation [74]. Indeed, during skin development, the primary cilium drives basal to spinous fate transition by regulating Notch signaling. Notably, ciliary NOTCH3 and the basal body-localized Presenilin2 are required for proper fate transition [74, 109] (Fig. 4c). These events occur independently and prior to Hedgehog-driven hair follicle morphogenesis. Notch pathway being a substantial governor of MuSC quiescence and self-renewal, it is tempting to speculate that the primary cilium could also contribute to its regulation [18, 110].

## Ciliated Cells Residing in Adult Skeletal Muscle

**Fig. 5:**
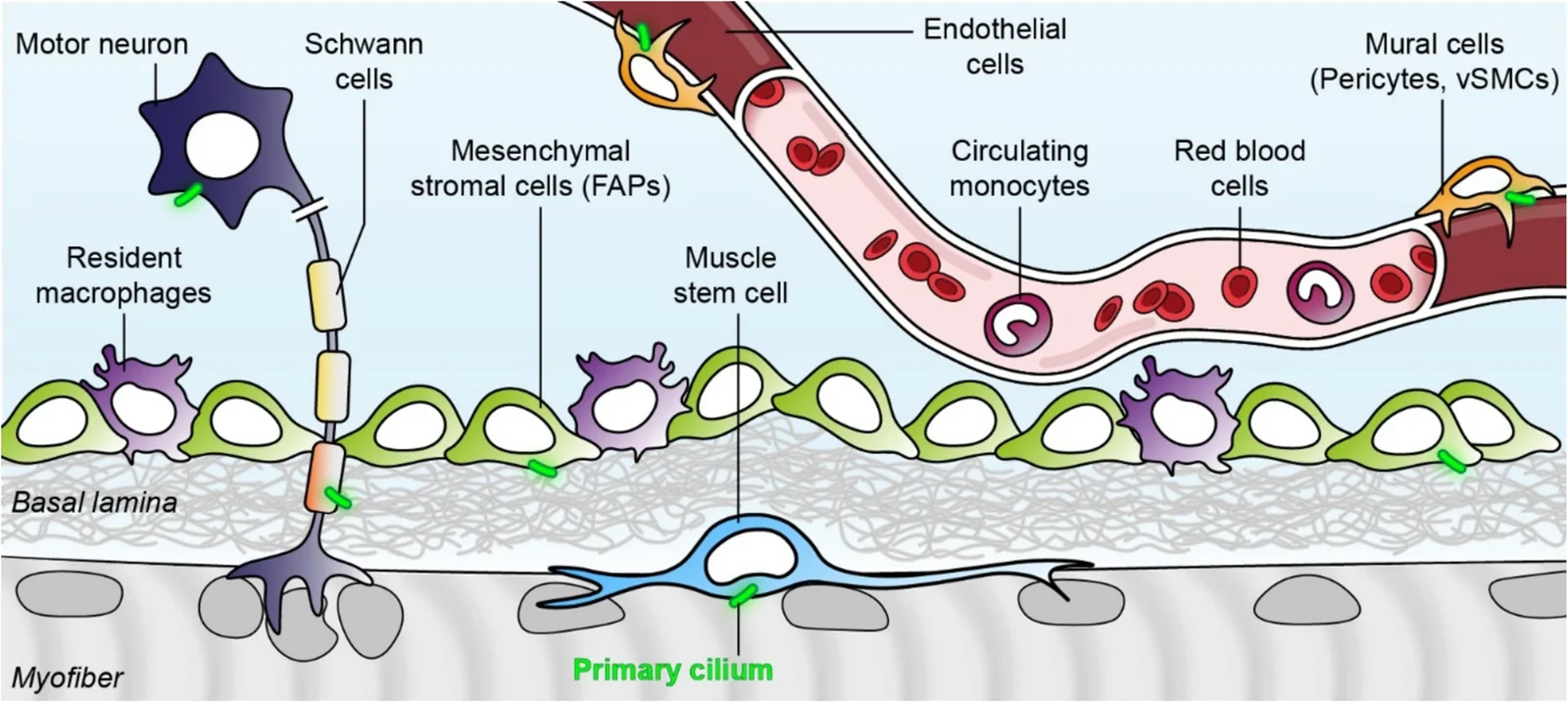
**Cellular niche of the muscle stem cell at steady-state.** Besides the myofiber and the basal lamina, the muscle stem cell niche comprises several other cell types that communicate and coordinate their behavior at homeostasis and during the muscle regeneration. First, there are the fibro-adipogenic progenitors (FAPs), a muscle-resident stem cell population in which the primary cilium drives differentiation toward a fibroblastic fate. The motor neuron, whose soma is located in the spinal cord, extends its axon to connect and innervate the myofiber to trigger its contraction. The axon is ensheathed by myelinating, non-myelinating and perisynaptic Schwann cells. Non-myelinating and perisynaptic Schwann cells have primary cilia, which are absent in myelinating Schwann cells. The muscle vascular component comprises ciliated endothelial and mural cells. Circulating red blood cells and immune cells, which patrol to respond rapidly to injury, lack primary cilia. Whether the muscle-resident macrophages harbor a primary cilium remains unknown

Skeletal muscle maintenance and regeneration require muscle stem cells (MuSCs) but also involve the coordinated responses of dozens of different cell types (Fig. 5) [111, 112]. Although many of these cells are ciliated (Table 2), the functions of primary cilia have been best characterized in the muscle-resident fibro-adipogenic progenitors (FAPs), largely through the original work of Daniel Kopinke’s laboratory [9, 114, 115].

**Table 2:** Roles of the primary cilium in homeostatic muscle-resident cells

Muscle-resident cell type	Function	Role(s) of the primary cilium
Muscle stem cells (MuSCs)	Allow skeletal muscle formation, regeneration and maintenance [1]	See Table 1 in this review
Fibro-adipogenic progenitors (FAPs)	Support skeletal muscle tissue maintenance and regeneration [113]	Cilia-mediated Hedgehog signaling controls FAP differentiation into adipocytes upon muscle injury [9, 114, 115].
Resident macrophages	Contribute to skeletal muscle tissue maintenance and regeneration [116]	N.D.
*Motor and sensory neurons	Allow muscle innervation and contractility	N.A. (*When present, the primary cilium typically extends from the neuronal soma, which is located outside the muscle tissue.)
Schwann cells	Form the myelin sheath surrounding axons of the peripheral nervous system, support axon maintenance and neuromuscular junction maturation [117]	While about 60% of non-myelinating and immature/pre-myelinating Schwann cells have primary cilia, they are absent in mature myelinating Schwann cells. Ciliary Hedgehog facilitates myelin sheath formation during the early phase of myelination [118]. At the neuromuscular junction, where the motor neuron axon connects to the myofiber, perisynaptic Schwann cells contain a primary cilium whose role remains undetermined [119].
Endothelial cells	Form a selective barrier between blood and tissues and support angiogenesis [120]	During vascular myogenesis in zebrafish, primary cilia of endothelial cells sense the blood flow, which triggers Notch activation and contributes to recruit mural cells to the newly formed blood vessels [99].
Mural cells (pericytes and vascular smooth muscle cells)	Support blood vessel development and stability [120]	Smooth muscle cells display a primary cilium that is enriched in mechanosensitive factors and controls cellular migration [34]. Although pericyte cilia have been identified in the carotid, their functional contribution remains to be investigated [121].
Tenocytes	Allow tendon homeostasis and repair [122]	At the myotendinous junction, mechanical loading triggers primary cilia disassembly in tenocytes, which in turn control tendon extracellular matrix turnover [96, 123]. Similarly, increased loading leads to cilia shortening and disassembly at tendon enthesis, overall diminishing mechano-induced Hedgehog signaling [124].

N.D.: not determined; N.A.: not applicable

FAPs are non-myogenic PDGFRα-expressing mesenchymal stem cells that represent the main population of mononuclear cells in skeletal muscle [113, 125–127]. They are multipotent cells capable of differentiating into fibroblasts or adipocytes. At homeostasis, FAPs reside in the interstitial space, where they primarily build extracellular matrix that helps maintain MuSC quiescence and provide trophic factors that promote myofiber growth. Although quiescent in uninjured muscle, FAPs rapidly expand upon muscle damage to promote extracellular matrix replacement, inflammatory cell infiltration, and MuSC-mediated regeneration [113]. Later in the regeneration process, they undergo massive apoptosis to return to steady-state levels. If only 10% of FAPs harbor a primary cilium in resting muscle, this proportion remarkably, but transiently, increases during the early stages of regeneration [9]. Interestingly, primary cilia allow FAPs to transduce Hedgehog signaling initiated by Desert Hedgehog (DHH) secretion from endothelial and Schwann cells upon injury [115]. DHH-binding to PTCH1 blocks ciliary GLI3 proteolytic processing, inducing *Gli1* and Hedgehog target gene expression alongside the upregulation of the *Tissue inhibitor of metalloproteinase 3*, which inhibits FAP conversion into adipocytes [9]. Therefore, Hedgehog signaling activation prevents muscle injury-induced adipogenesis. Consequently, ciliary Hedgehog activation indirectly promotes skeletal muscle repair by increasing the density and size of regenerating myofiber at the expense of intramuscular fat accumulation [9, 114].

As observed in the MuSC population, *Gli1* expression also identifies a discrete subset of FAPs [71, 72, 128]. *Gli1* + FAPs exhibit enhanced proliferative capacity upon tissue damage and a shifted fate towards fibrogenesis rather than adipogenesis, compared to their *Gli1*- counterparts [128]. They express increased levels of pro-myogenic and anti-adipogenic factors, thus promoting MuSC expansion while inhibiting intramuscular adipogenesis. Importantly, GLI1 expression does not only mark this subpopulation of FAPs but actively restrains their expansion and adipogenic differentiation [72]. As a consequence, *Gli1* knockout in FAPs strongly impairs muscle regeneration. Aside from Hedgehog signaling, FAP primary cilium regulates other signaling pathways that control their fate. In a mouse model lacking *Trichoplein* (*Tchp*^−/−^), an inhibitor of ciliogenesis, FAPs display elongated cilia that halt adipogenic differentiation during muscle regeneration [129, 130]. Mechanistically, ciliary elongation impairs the adipogenesis-induced accumulation of lipid rafts/caveolae around the ciliary base, which consequently suppresses the Insulin/Akt pathway [129, 131]. Concomitantly, length elongation of FAP primary cilia in *Tchp*^−/−^ mice stimulates ST2/c-Jun N-terminal kinase 1/2 signaling axis, triggering secretion of the pro-myogenic cytokine, Interleukin-13 [129]. Altogether, these studies highlight the essential role of FAP primary cilium in controlling intramuscular adipogenesis and promoting muscle regeneration.

## Methodological Challenges to Studying Primary Cilia in Skeletal Muscle

**Fig. 6:**
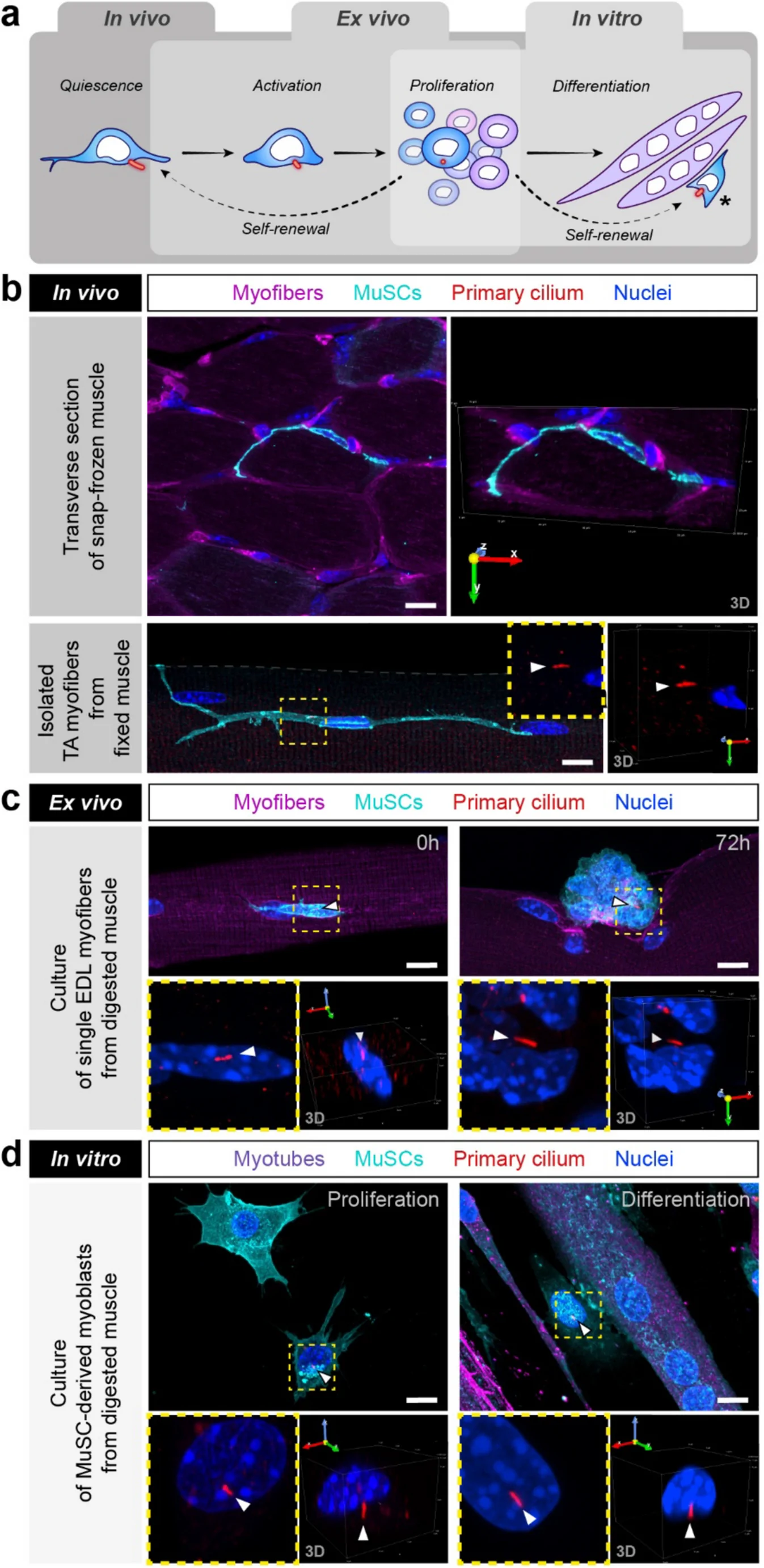
**Current methods to study cilia dynamics in muscle stem cells.** **a)** Schematic of the muscle stem cell (MuSC) lineage. In vivo assays allow the analysis of MuSCs at all stages of their lineage, whereas ex vivo approaches only permit the observation of MuSCs from quiescence exit to early differentiation and self-renewal. In vitro MuSC-derived myoblasts are mainly used for proliferation and differentiation assays. Of note, upon myoblast differentiation into multinucleated myotubes, a population of non-cycling and mononucleated cells appears alongside myotubes. These cells, called reserve cells (asterisk), can serve as a proxy for self-renewing cells. In the following panels (b-d), we used a tamoxifen-inducible mouse model, in which MuSCs are labeled by membrane-localized GFP (cyan) upon tamoxifen treatment, while all the other cells, including the myofibers, are marked by membrane-localized tdTomato (magenta) [21]. The primary cilium is stained with an anti-ARL13b antibody (red). Nuclei are labeled with DAPI (blue). All scale bars, 10 μm. **b)** In vivo. Representative immunofluorescence of homeostatic MuSC. **Top panel**: Transversal section of snap-frozen *tibialis anterior* (TA) skeletal muscle, showing a quiescent MuSC (cyan) that enwraps a myofiber (magenta), in standard 2D (left) or 3D-reconstructed view (right). The primary cilium is rarely visible. **Bottom panel**: Ciliated MuSC on a single myofiber isolated from fixed TA muscle shown in 2D (left, with inset magnifying the boxed region to highlight the primary cilium, arrowhead) and as a 3D reconstruction (right). **c)** Ex vivo. Immunofluorescence of MuSCs on myofibers isolated from enzymatically digested *extensor digitorum longus* (EDL) muscle, either fixed directly post-isolation (0 h, left) or after 72 h of culture (right). At 0 h, MuSC maintains a primary cilium but the quiescent projections are drastically reduced. At 72 h, MuSCs have proliferated into clusters of differentiated cells and ciliated self-renewing MuSCs. Boxed regions are magnified below in 2D and 3D reconstruction with arrowheads indicating the primary cilium. **d)** In vitro. Immunofluorescence pictures of MuSC-derived myoblasts isolated from all hindlimb skeletal muscle, either cultured in proliferating conditions (left) or in differentiation medium (right). Myotubes are stained with an anti-myosin heavy chain (MyHC) antibody. In proliferation, undifferentiated myoblasts rarely display a primary cilium. Upon differentiation, the self-renewing reserve cells are mononucleated, do not express myosin heavy chains and exhibit a primary cilium. Boxed regions are magnified below in 2D and 3D reconstruction with arrowheads indicating the primary cilium

It is now clear that primary cilia are present in many muscle-resident cell types, where they contribute to skeletal muscle development and homeostasis [132]. However, despite major advances in microscopy and imaging that have made primary cilia much easier to study [133, 134], progress in defining their function in muscle-resident cells, and especially in muscle stem cells (MuSCs), remains limited. This likely reflects both the biological complexity of muscle tissue and the methodological constraints inherent to current experimental systems. Most of our knowledge of MuSC biology has come from the combined analysis of in vivo skeletal muscle tissue, ex vivo culture of single muscle fibers, and in vitro culture of MuSC-derived primary myoblasts from both mouse and human biopsies (Fig. 6a) [135].

### Visualizing Muscle Stem Cell Cilia in Situ

Skeletal muscle tissue is particularly difficult to analyze because of its complex and heterogeneous organization. It is mainly composed of multinucleated myofibers, surrounded by a dense extracellular matrix, and it comprises many cell types, among which MuSCs themselves are rare and highly sensitive to tissue handling. This is why transversal muscle sections are commonly used to analyze MuSC behavior within their intact niche in vivo (Fig. 6b) [136]. However, visualizing MuSC primary cilia in such sections is very challenging because of geometric constraints, and the usual 10 μm thickness of the sections limits visualization of the entire MuSC. This becomes even more difficult in sections of regenerating muscles, where many cells infiltrate the tissue and primary cilia are dynamically assembled and disassembled on activated and proliferating MuSCs. The development of tissue-clearing protocols has enabled reconstruction of the three-dimensional structure of muscle tissue at homeostasis and during regeneration without disrupting the MuSC niche [21]. However, MuSC labeling in cleared tissue samples requires fluorescent reporters, which would also be necessary to visualize their primary cilia. For instance, the Cilia-Glow mouse, in which all primary cilia are labeled with mCherry, allowed analysis of cilia dynamics during adipogenesis in situ [137, 138]. Yet these methods only provide static imaging of MuSCs in vivo. In the future, combining MuSC and cilia reporters could allow live tracking, *via* intravital imaging, of MuSC cilia dynamics in homeostatic, regenerative or pathophysiological contexts [139, 140].

The simplest way to overcome the constraining environment and improve visualization of MuSCs is to isolate bundles or individual myofibers from fixed muscles [141, 142]. These protocols preserve the architecture of the quiescent MuSC – in particular its cytoplasmic projections – and facilitate visualization of primary cilia without the need for fluorescent reporters (Fig. 6b). However, they are not applicable to regenerating muscle in which myofibers are lost or rendered extremely fragile. Most importantly, they fail to provide a dynamic overview of MuSC behavior as they progress into the myogenic lineage.

### Tracking Muscle Stem Cell Cilia Ex Vivo

Ex vivo approaches aim to analyze muscle stem cell (MuSC) behavior by using isolated floating myofibers as a support. They first require the dissection of skeletal muscle from tendon to tendon in order to preserve myofiber integrity and their associated MuSCs [143]. Consequently, these approaches rely mainly on mouse tissue samples [144], although human muscle fibers have already been successfully used to assess MuSC dynamics [145]. Once dissected, the muscle undergoes enzymatic digestion and mechanical dissociation to release individual myofibers. Single myofibers can then be fixed immediately, which allows the observation of the quiescent features of MuSCs, albeit to a lesser extent than in myofibers isolated from fixed muscle (Fig. 6c, 0 h culture), owing to niche disruption during the digestion step [10, 11, 15, 44, 49].

Isolated single myofibers can also be cultured for several days in order to track MuSC activation, motility, proliferation and fate (early differentiation and self-renewal, Fig. 6c, 72 h culture) [10, 11, 139]. This method is highly powerful for precisely deciphering the cellular and molecular consequences of genetic modification or pharmacological treatment on MuSCs specifically. While the enzymatic-based digestion of the extracellular matrix partially triggers MuSC activation, it also greatly improves protein accessibility for immunostaining and high-resolution of primary cilia imaging as MuSC progress into their lineage [7, 10, 11, 66]. Further development in live imaging and combination of cilia- and MuSC-fluorescent reporters should provide a better understanding of primary cilia dynamics during MuSC lineage progression [139, 140].

### Mimicking Muscle Stem Cell Biology In Vitro

The most commonly used method is the monolayer culture of muscle stem cell (MuSC)-derived myoblasts in vitro. Differentiation assays generate myotubes (immature myofibers) as well as non-cycling ciliated cells termed “reserve cells”, which somewhat resemble self-renewing MuSCs [146] (Fig. 6a). These latter can serve as a proxy to compare cilia dynamics in proliferating myoblasts and self-renewing reserve MuSCs [11] (Fig. 6d). Mouse MuSC-derived myoblasts support in vitro expansion for a few passages, allowing high-throughput analysis of proliferation, differentiation and fusion capacities. However, the low ciliation of proliferating myoblasts combined with the scale of this organelle renders bulk analysis difficult using classical biochemical approaches. Targeted methods such as biotin proximity labeling assays should offer further strategies to overcome these technical limitations and have already been applied successfully to *Xenopus* and mouse neuronal cilia [30, 147, 148].

Human muscles being of much bigger size than their murine counterparts, patient muscle biopsies can be processed to isolate and amplify MuSCs in vitro [14]. Because human myoblasts can only be kept in culture for a very limited number of passages, they are usually immortalized for high-throughput behavioral analysis but immortalization can tamper with cilia dynamics [149]. This limitation impedes the study of primary cilia dynamics and signaling in patients suffering from myopathies or ciliopathies, especially when it comes to cilia function in quiescent MuSCs. Yet, bringing awareness to the presence and role of the primary cilium might encourage scientists to quantify cilia dynamics in patient cells in vitro, alongside the canonical readouts of proliferation, fusion index, and cell fate.

Specifically, the development of three-dimensional culture systems and biomaterial-based scaffolds offers a promising avenue to better recapitulate the native MuSC niche in vitro. Engineered hydrogels [150, 151], decellularized muscle matrices [152], and synthetic scaffolds with tunable stiffness and composition [153–155] can mimic key mechanical and biochemical cues of the basal lamina, which are largely absent in standard myoblast cultures. These approaches have already proven valuable for studying MuSC self-renewal and fate decisions, and could similarly provide a more physiological context to investigate primary cilia assembly, disassembly, and signaling. Of note, 3D systems allow co-culture of MuSCs with other niche components (such as myofibers, fibro-adipogenic progenitors, endothelial or neural cells) [156], and could thereby better preserve the signaling environment required for proper ciliary function, while remaining compatible with live imaging and high-throughput screening. Together, these 3D culture platforms could bridge the gap between the simplicity of 2D myoblast cultures and the complexity of in vivo skeletal muscle tissue. They will ease the analysis of primary cilia dynamics throughout MuSC lineage progression, including how it is regulated by the mechanical and biochemical environment.

## Conclusion

Over the last decade, the primary cilium has emerged as a microtubule-based structure that regulates muscle stem cell function. Now a well-established marker of quiescence, the primary cilium maintains muscle stem cell (MuSC) dormancy under homeostatic conditions, at least by repressing the Hedgehog pathway [11, 66]. Yet because not all quiescent MuSCs are ciliated, the primary cilium cannot be reduced to a marker of quiescence. It appears instead to define a depth of quiescence, acting as a gatekeeper that integrates biochemical and mechanical cues from acutely injured or exercised muscle to adjust MuSC activation to tissue demand [7, 67].

This sensing function persists during muscle regeneration, where ciliary Hedgehog signaling also regulates MuSC expansion and fate decisions [7, 11, 51, 66, 67]. Consequently, cilia and/or Hedgehog signaling disruption impairs exercise-induced muscle hypertrophy and normal regeneration. Altogether, these findings establish that the primary cilium is a dynamic structure whose assembly, disassembly, and signaling output are remodeled at each step of the myogenic lineage, allowing MuSCs to respond appropriately to their local environment. The precise nature of the extracellular cues sensed by the cilium in a pro-myogenic context remains incompletely defined. Recent developments in biosensors, optogenetic systems, and proximity labelling tools will provide valuable insights into identifying new pathways controlling MuSC quiescence, activation, proliferation, fate, and self-renewal [27].

Since most of this knowledge comes from mouse genetic models, its relevance to humans still needs to be clarified. This is all the more true given that inherited cilia-related disorders, referred to as ciliopathies, affect many different tissues and organs including the brain, eye, kidney, liver, bone, and nervous system, yet primary muscle symptoms have generally not been described [157]. When muscle-related defects occur, for instance hypotonia or ataxia as observed in Joubert syndrome, they are usually considered secondary to brain or central nervous system involvement [158]. Since skeletal muscle fibers themselves lack a primary cilium, it is not surprising that muscle tissue has been relatively overlooked in ciliopathy patients. As such, muscle-specific defects during exercise or injury – two processes that, in mice, rely on intact MuSC ciliary signaling – have not been examined, even though they could plausibly contribute to progressive muscle dysfunction if MuSCs fail to maintain or repair muscle over time. Importantly, the invasive nature of muscle biopsy further limits characterization in patients who lack overt muscle phenotypes, leaving this population essentially uncharacterized at the MuSC level. This is especially relevant as physical exercise emerges as a complementary approach alongside treatments to improve symptoms and quality of life in patients with ciliopathies [159]. A better understanding of MuSC defects will therefore be important. Likewise, ciliary defects in aged human MuSCs remain to be investigated to determine whether they contribute to the age-related decline in stem cell competence [7].

Ciliary defects also remain largely unexplored in neuromuscular disorders, which refer to conditions affecting directly the muscle fiber, or indirectly through problems with the peripheral nervous system and/or the neuromuscular junction. This can be partly explained by the fact that myofibers are devoid of primary cilia. Interestingly, a systematic review of the literature has identified muscular dystrophies in which the MuSC function is primarily affected [160]. This portfolio of “satellite cell-opathies” keeps increasing as the evaluation of MuSC function in these disorders improves [161]. As the methodology and tools for studying MuSC primary cilia expand, it may be reasonable to think that the biology of MuSC primary cilia in mice will be translated soon to humans. Primary myoblasts derived from patient biopsies should offer an optimal platform to assess ciliation rate and Hedgehog pathway activity by immunostaining for ARL13B and GLI3, for instance.

The clinical relevance of MuSC cilia biology is perhaps most clearly illustrated by rhabdomyosarcoma, a pediatric soft tissue sarcoma that morphologically and transcriptionally resembles developing skeletal muscle despite a non-myogenic cell origin [51, 162, 163]. Cilia dysregulation is now directly implicated in rhabdomyosarcoma pathogenesis through at least two convergent mechanisms. First, recent work has proposed that primary cilia define the cell of origin of rhabdomyosarcoma [163]. Transient presence of primary cilia in early endothelial cell progenitors defines a window of competency for Hedgehog signaling activity that progressively switches off during endothelial differentiation. This competency further determines rhabdomyosarcoma transformation potential [163]. Second, in myogenic rhabdomyosarcoma cell lines, cilia assembly and Hedgehog signaling are perturbed compared to normal MuSC-derived myoblasts. This deregulation is thought to sustain aberrant proliferation, hindering cell cycle exit and proper differentiation [51]. Together, these findings warrant direct investigation of the primary cilium and ciliary Hedgehog signaling as therapeutic targets in rhabdomyosarcoma [162].

As the primary cilium emerges as a signaling hub that governs MuSC function, from quiescence to regeneration to disease, it raises a new set of questions with direct relevance to both muscle physiology and pathology. Advances in new technologies over the coming years will undoubtedly provide critical insights that will help further elucidate the role of primary cilia in muscle stem cell biology, and translate these findings into insights relevant to human muscle disease.

## Data Availability

No datasets were generated or analysed during the current study.

## Abbreviations

ADCY6: Adenylyl Cyclase 6
AURKA: Aurora kinase A
CALCR: Calcitonin receptor
CDK4/6: Cyclin-dependent kinase 4/6
CK1ε: Casein kinase 1ε
COLV: Collagen V
DHH: Desert Hedgehog
FAPs: Fibro-Adipogenic Progenitors
GLI: Glioma-associated oncogene
GLI3FL: GLI3 full-length
GLI3R: GLI3 repressor
GPCR: G protein-coupled receptor
GSK3β: Glycogen synthase kinase 3β
HDAC6: Histone deacetylase 6
IFT: Intraflagellar transport proteins
MuSCs: Muscle Stem Cells
MyHC: Myosin heavy chains
NPHP4/9: Nephrocystin 4/9
PAX7: Paired-box protein 7
PKA: cAMP-dependent protein kinase A
PLK1: Polo-like kinase 1
PTCH1: Patched 1
SAG: Smoothened agonist
SMO: Smoothened
TAZ: Transcriptional co-activator with PDZ-binding motif
Tchp: Trichoplein
TubA: Tubastatin A
YAP: Yes-associated protein 1
